# Bacterial microbiota diversity and composition in red and white wines correlate with plant-derived DNA contributions and botrytis infection

**DOI:** 10.1038/s41598-020-70535-8

**Published:** 2020-08-14

**Authors:** Alena M. Bubeck, Lena Preiss, Anna Jung, Elisabeth Dörner, Daniel Podlesny, Marija Kulis, Cynthia Maddox, Cesar Arze, Christian Zörb, Nikolaus Merkt, W. Florian Fricke

**Affiliations:** 1 Department of Microbiome Research and Applied Bioinformatics, Institute of Nutritional Sciences, University of Hohenheim, Stuttgart, Germany; 2 Department of Plant Quality and Viticulture, Institute of Crop Science, University of Hohenheim, Stuttgart, Germany; 3 Institute for Genome Sciences, University of Maryland School of Medicine, Baltimore, MD USA; 4 Present Address: Personal Genome Diagnostics, Baltimore, MD USA; 5 Present Address: Ring Therapeutics, Cambridge, MA USA

**Keywords:** Computational biology and bioinformatics, Microbiology, Plant sciences, Environmental sciences

## Abstract

Wine is a globally produced, marketed and consumed alcoholic beverage, which is valued for its aromatic and qualitative complexity and variation. These properties are partially attributable to the bacterial involvement in the fermentation process. However, the organizational principles and dynamic changes of the bacterial wine microbiota remain poorly understood, especially in the context of red and white wine variations and environmental stress factors. Here, we determined relative and absolute bacterial microbiota compositions from six distinct cultivars during the first week of fermentation by quantitative and qualitative 16S rRNA gene amplification and amplicon sequencing. All wines harboured complex and variable bacterial communities, with *Tatumella* as the most abundant genus across all batches, but red wines were characterized by higher bacterial diversity and increased relative and absolute abundance of lactic and acetic acid bacteria (LAB/AAB) and bacterial taxa of predicted environmental origin. Microbial diversity was positively correlated with plant-derived DNA concentrations in the wine and *Botrytis cinerea* infection before harvest. Our findings suggest that exogenous factors, such as procedural differences between red and white wine production and environmental stress on grape integrity, can increase bacterial diversity and specific bacterial taxa in wine, with potential consequences for wine quality and aroma.

## Introduction

Wine is a popular alcoholic beverage, which is cherished for its versatile aroma and complexity worldwide. Although it is globally produced and marketed, regional wine varieties include prominent, often historic and legally protected, geographic pedigrees and appellations. While specific wine “terroirs” or phenotypic characteristics have been associated with quantifiable molecular markers, such as chemical and metabolite profiles^1,2^ and sensory attributes^3–5^, many of the underlying mechanisms for the development of colour, aroma and flavour variations remain poorly understood. The most important intrinsic and extrinsic factors that have been identified include grape-specific differences in secondary microbial metabolite diversity and composition; soil, weather, and climate; geological conditions and environmental stress factors; viticulture and the winemaking process itself^6–8^.

As wine colour, aroma and flavour are substantially affected by microbial fermentation of the grape must, the taxonomic composition and functional repertoire of the wine microbiota, as well as its dependence on environmental influences, are of great interest^9^. Besides eukaryotic yeasts as the drivers of alcoholic fermentation, bacteria are known to contribute to malolactic acid fermentation and other metabolic processes^10,11^. The diversity, composition and biogeography of the fungal and bacterial microbiota of wine has been illustrated by several recent cultivation-independent, high-throughput amplicon sequencing studies^11–17^. Differences in wine microbiota profiles have been associated with the grapevine, including distinct cultivars^14,15,18^, plant organs^17,19^, and vintages^14^. Environmental sources of microbes in wine have been identified as plant leaves and roots^17,19^, soil^15,17,20^, and the winery setting^21^. Not surprisingly, the wine microbiota is affected by the fermentation type, i.e. spontaneous or natural fermentation as opposed to inoculated fermentation with yeast starter cultures^22,23^, and fungal infections, including sour rot and Botrytis bunch rot^23,24^. Interestingly, bacterial microbiota differences between red and white wines have received limited recognition, despite distinct production processes and well-characterized metabolic and aromatic profile differences. For white wine production, red or white grapes are crushed and pressed and only the clarified juice is used for fermentation, whereas a mixture of juice with skin and seeds from crushed red grapes is used for red wine fermentation^25^. As a result, red wines are characterized by increased concentrations of secondary plant metabolites, such as flavonoids, including anthocyanins, flavonols and flavanols; non-flavonoids, including hydroxybenzoic and hydroxycinnamic acids and stilbenes; and other phenolic compounds, many of which have been associated with health-promoting benefits^26,27^.

In order to improve our understanding of the composition, organization and temporal dynamics of the red and white wine bacterial microbiota, we determined relative and absolute microbiota compositions from six distinct cultivars during the first week of fermentation by 16S rRNA gene amplification and amplicon sequencing. All wines were found to harbour complex bacterial communities with substantial variations between red and white wines, distinct cultivars and even separate fermentations from the same cultivar. Increases or decreases in the relative abundance of specific bacterial taxa during the fermentation were associated with changes in total bacterial concentrations and the observed differences between time points, wine types and cultivars were most strongly correlated with microbial diversity. Variations in diversity could be attributed to plant-derived DNA contributions and the influence of environmental stress factors, such as *Botrytis cinerea* fungal burdens of grapes before harvest. Our findings point to exogenous factors contributing to bacterial microbiota diversity in wine with both potentially desirable and undesirable consequences for wine quality and aroma.

## Results

### Wine sampling and microbiota analysis overview

Metagenomic DNA was isolated from seven batches of fermenting wines, including two red wines (Regent/REG, Schwarzriesling/SCH) and four white wines (Helios/HEL, Merzling/MER, Seyval/SEY, Bacchus/BAC) (Table 1). Two independent BAC fermenting batches were included, BAC1 from regular grapevines as opposed to BAC2, which had been treated with a commercial combination of insect attractant and insecticide (Combi-protec, Belchim; BAC2). All grapes were grown on the same vineyard, harvested within three weeks, processed and fermented in close proximity at the same winery at the viticulture unit of the University of Hohenheim, Stuttgart, Germany. Available metadata for all wines included metabolic differences between batches at the beginning and/or end of the fermentation period of 14 days (pH, total acid, alcohol and sugar content), changes during fermentation (°Brix/must weight) and rates of infection of grapes with the fungal plant pathogen *Botrytis cinerea* at the time of harvest (Supplementary Table S1). Longitudinal samples were collected 3–4 times daily for the first three days and once per day for each remaining day during the first week of fermentation. (Table 1). The first sample was collected within two hours after grape pressing and before inoculation with commercial yeast starter cultures (Supplementary Table S1). Bacterial taxonomic microbiota compositions were determined by 16S rRNA gene amplicon sequencing, resulting in 5.38 million taxonomically assigned sequences from a total of 84 samples. Of these, ~ 58% were classified as plant-derived reads, i.e. chloroplast and mitochondrial sequences, which showed grape vine (*Vitis vinifera*) and rootstock (*Vitis riparia*) as the closest matches in public databases (Supplementary Table S2). For most analyses, unless indicated otherwise, plant-derived reads were removed, resulting in a dataset of 2.21 million sequences. After rarefaction to 3,500 reads per sample, 520 distinct bacterial species equivalent or operational taxonomic units (OTUs) were identified. Quantitative 16S rRNA gene amplifications were carried out at three time points, on days 0, 3 and 7, and used to determine absolute bacterial abundances or bacterial loads, i.e. 16S rRNA gene copies per millilitre of wine. Summary statistics of sequencing output, taxonomic compositions and quantitative analyses are shown in Supplementary Tables S3 and S4.

**Table 1:** Sample and metadata overview.

	Sampling schedule (Days/hours)												*B. cinerea* (%)^a^	Total acid (g/L)	
**Day**	**0**	**1**			**2**			**3**	**4**	**5**	**6**	**7**	**0**	**0**	**7**
**Hour**	0^b^	2	8	15	23	31	39	50^b^	73	97	121	145^b^	0	0	145
**Red wines**															
Regent/REG	x	x	x	x	x	x	x	x	x	x	x	x	5	7.5	7.3
Schwarzriesling/SCH	x	x	x	x	x	x	x	x	x	x	x	x	30	8.8	7.6
**White wines**															
Merzling/MER	x	x	x	x	x	x	x	x	x	x	x	x	5	7.7	6.7
Seyval/SEY	x	x	x	x	x	x	x	x	x	x	x	x	30	7.3	6.5
Helios/HEL	x	x	x	x	x	x	x	x	x	x	x	x	40	9.6	8.1
Bacchus 1/BAC1	x	x	x	x	x	x	x	x	x	x	x	x	15	6.7	6.1
Bacchus 2/BAC2	x	x	x	x	x	x	x	x	x	x	x	x	15	6.4	5.9

The first sample of each wine was collected immediately before inoculation with the yeast starter culture. BAC2 grapes were treated with combi-protec with insecticide SpinTor (Belchim Crop Protection).

^a^Infectious burden as percentage of affected grapes.

^b^Samples selected for qPCR.

### Differences in bacterial microbiota concentration, diversity and composition between red and white wines

**Figure 1:**
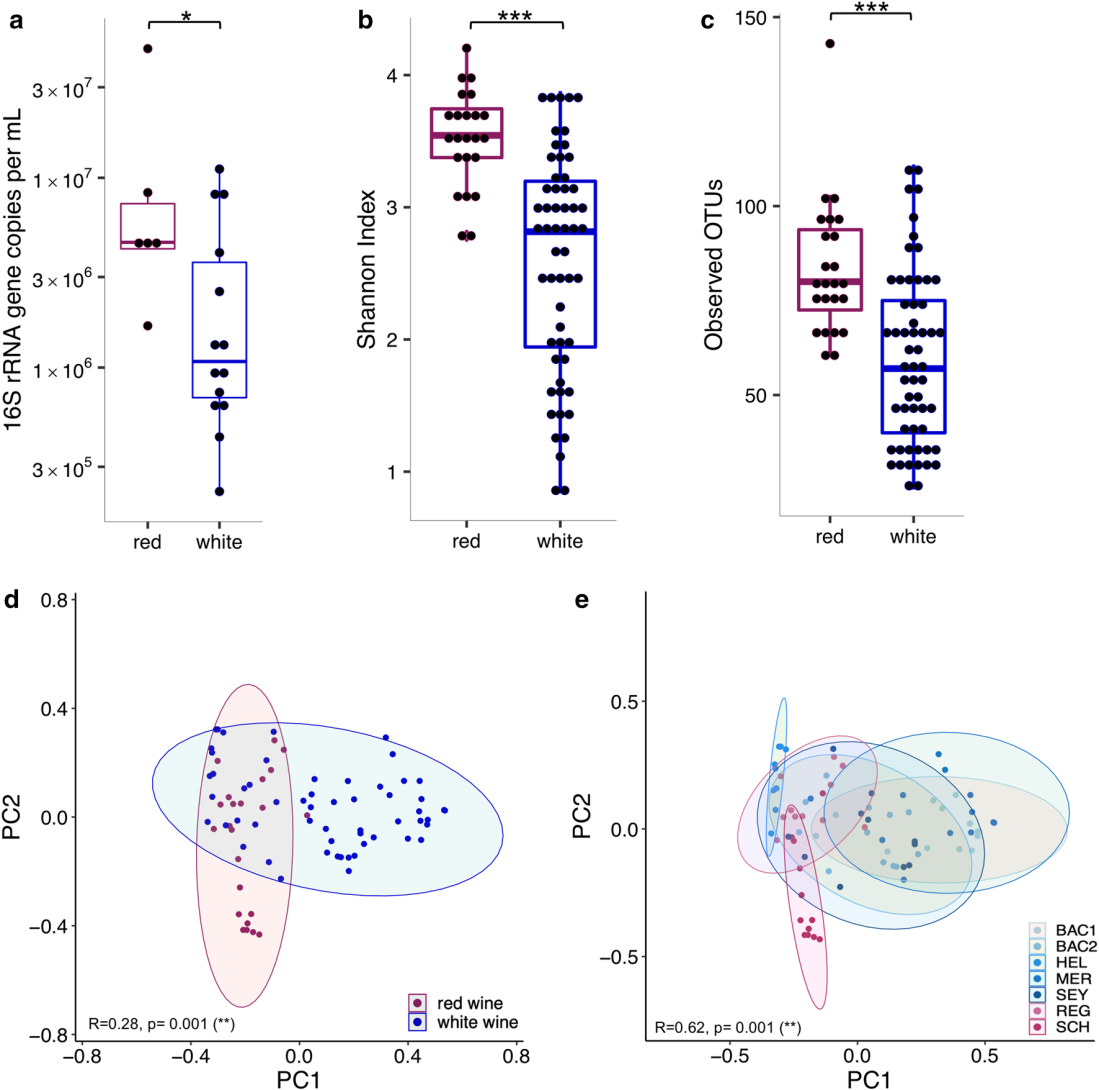
Red and white wine bacterial microbiota comparison. Red and white wine samples across all time points were compared based on 16S rRNA gene copy numbers per mL (**a**), and bacterial microbiota diversity, based on Shannon Index (**b**) and observed OTUs (**c**), as well as taxonomic composition, based on Bray–Curtis dissimilarity (**d**). Taxonomic distances were also compared for all red and white wines separately (**e**). Significance was calculated based on Wilcoxon rank-sum test, corrected with the Benjamini–Hochberg procedure (**a**–**c**) and ANOSIM with 999 permutations (**d**, e) with *p < 0.05, **p < 0.01, ***p < 0.001. Sample numbers: n_red wine_ = 23, n_white wine_ = 56, n_BAC1_ = 12, n_BAC2_ = 12, n_HEL_ = 9, n_MER_ = 12, n_SEY_ = 11, n_SCH_ = 11 and n_REG_ = 11.

Compared to white wines, red wines harboured increased bacterial loads of larger taxonomic diversity, as determined based on higher concentrations of bacterial 16S rRNA genes (Fig. 1a), increased alpha-diversity (Fig. 1b) and more observed OTUs (Fig. 1c). Taxonomic bacterial microbiota compositions showed minor but significant differences between red and white wines (Fig. 1d), as well as between separate red and white wine batches (Fig. 1e).

**Figure 2:**
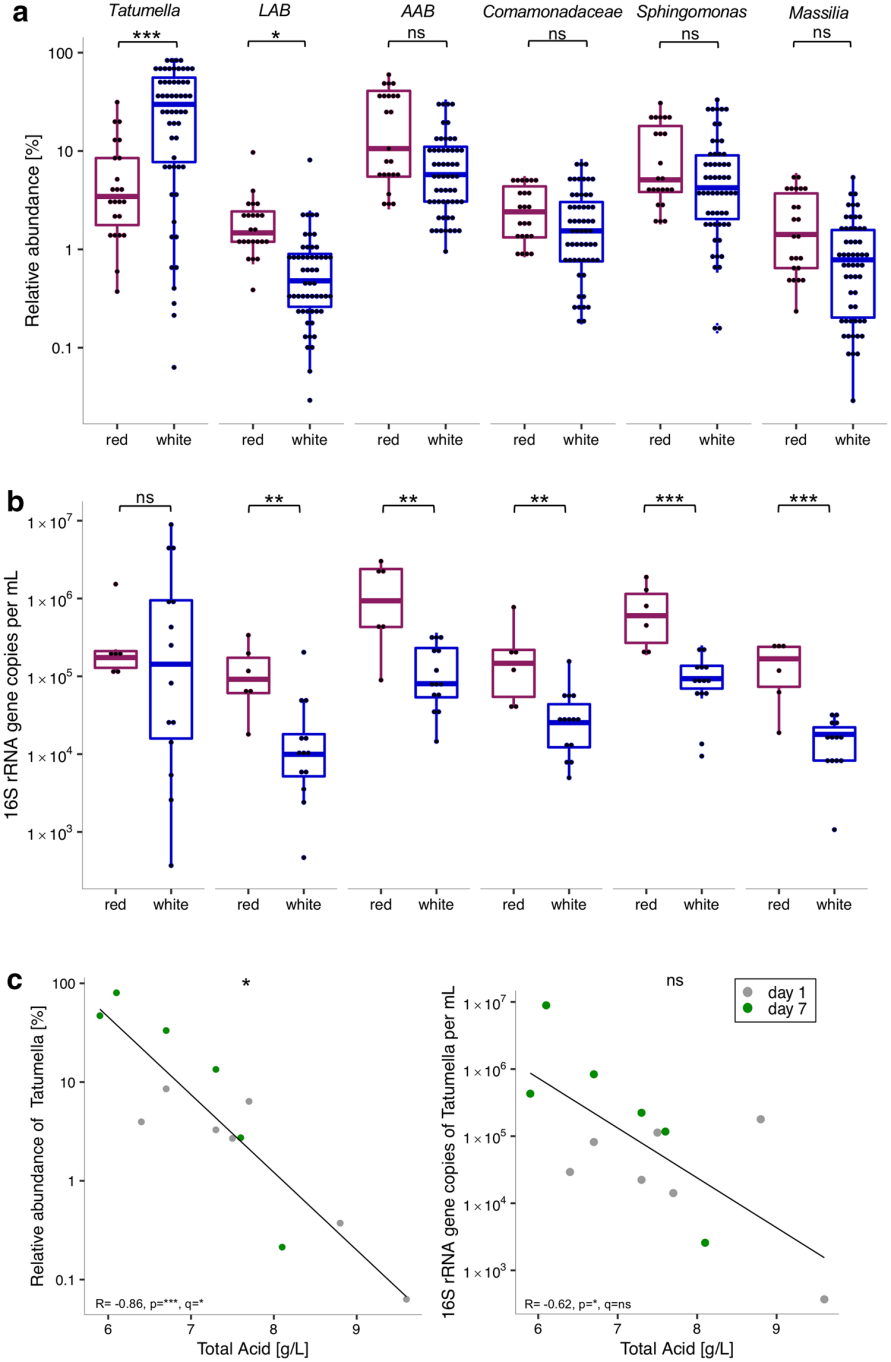
Bacterial groups with differential relative (**a**) and absolute (**b**) abundance in red and white wines and association with total acid content in wine (**c**). The relative abundances, based on 16S rRNA gene amplicon sequencing (**a**) and absolute abundances, based on 16S rRNA gene copy number concentrations (**b**) in red and white wines were compared for the proteobacterial genus *Tatumella*, lactic acid bacteria (LAB), acetic acid bacteria (AAB) and the proteobacterial family *Comamonadaceae* and genera *Sphingomonas* and *Massilia*. Relative and absolute abundance of *Tatumella* and total acid content (g/L) were negatively correlated, based on available metadata (**c**). Significance was calculated based on ALDEx analysis with 128 DMCs (**a**, **b**) and Spearman’s rank correlation test (**c**), all corrected with the Benjamini–Hochberg procedure, with ns = not significant, *p/q < 0.05, **p/q < 0.01, ***p/q < 0.001. Effect sizes for ALDEx analyses in (**a**): *Tatumella* = 0.87, LAB = − 0.47, AAB = − 0.14, *Comamonadaceae* = 0.21 *Sphingomonas* = 0.21, *Massilia* = − 0.11.

The genus *Tatumella* from the phylum *Proteobacteria* was the most abundant bacterial taxon across all samples, both in terms of relative (26 ± 3%) and absolute abundance (10^6^ ±  5*10^5^ 16S rRNA gene copies/mL). The latter was calculated as the taxon-specific fraction of the total bacterial 16S rRNA gene copy number per sample. While the relative abundance of *Tatumella* was higher in white compared to red wines (Fig. 2a), there was no difference in absolute abundance between both wine types (Fig. 2b), suggesting higher loads of additional other bacteria in red wines as the source of reduced relative abundances of *Tatumella*, which demonstrates the utility of comparing both metrics for microbiota analysis. *Tatumella* relative and absolute abundance were negatively correlated with total acid content (Fig. 2c), which was the only significant association between individual bacterial taxa and the available metabolic metadata after correcting for multiple testing.

The groups of acetic acid bacteria (AAB) and lactic acid bacteria (LAB) are often used to refer to bacteria that can negatively and positively affect the wine fermentation process, aroma and quality^10,11^. For our analysis, we combined all OTUs from those genera typically assigned to AAB (i.e. *Acetobacter*, *Gluconacetobacter*, and *Gluconobacter*) and LAB (*Lactobacillus*, *Oenococcus*, and *Lactococcus*) and found LAB to be overrepresented in red compared to white wines, both in terms of relative (Fig. 2a) and absolute abundance (Fig. 2b). Increased bacterial loads in red wine were also found for AAB and the family *Comamonadaceae*, the genera *Sphingomonas* and *Massilia* (abbreviated from here on as CSM, Fig. 2b).

### Changes of the bacterial wine microbiota during fermentation

**Figure 3:**
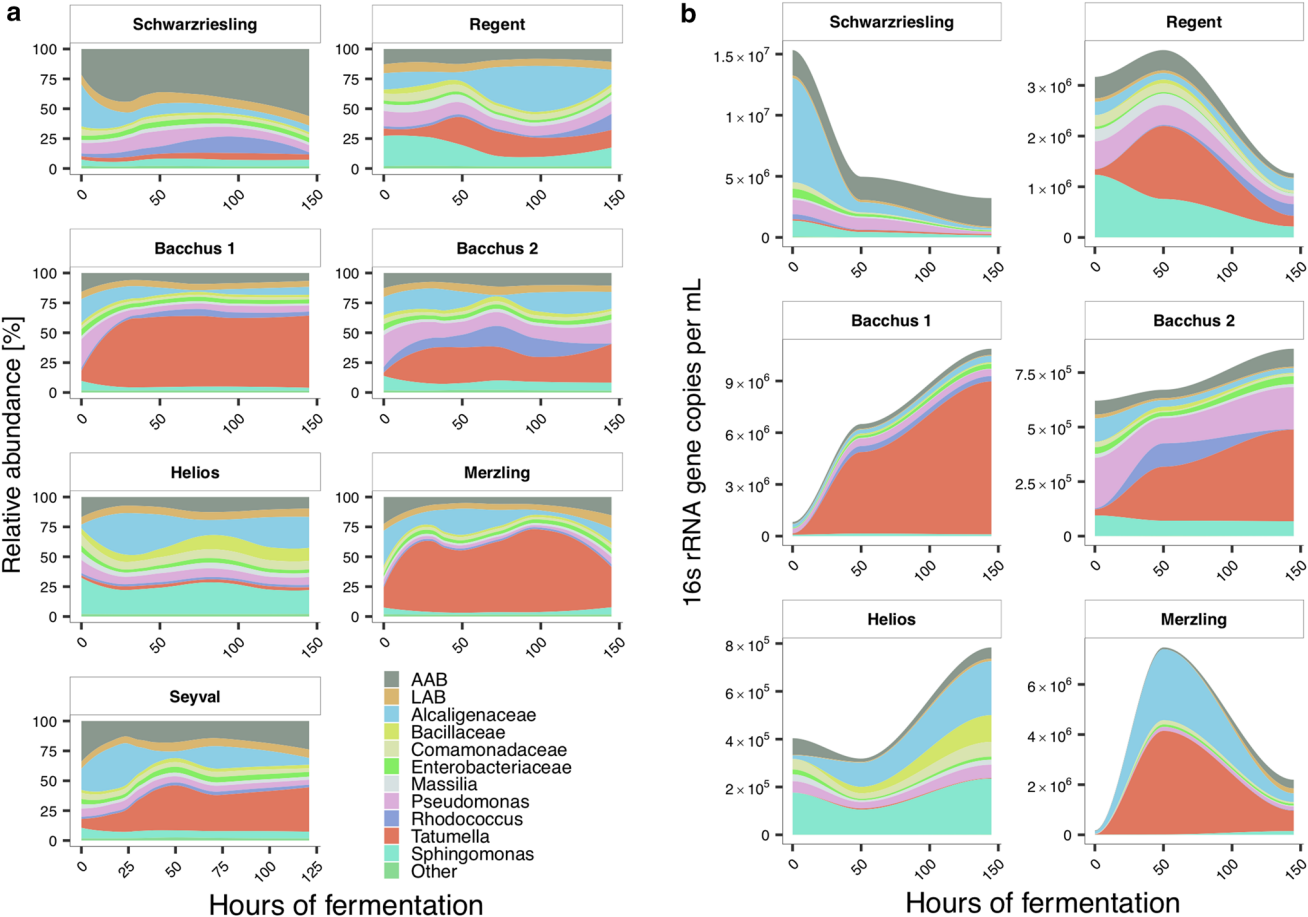
Relative (**a**) and absolute (**b**) bacterial microbiota composition changes during fermentation. For the visualization an abundance threshold was set of either ≥ 1% in all samples or ≥ 5% in at least one sample. The eleven most abundant assigned taxa on the genus level are shown in the figure. Locally weighted regression was used to smooth relative abundances over time.

**Figure 4:**
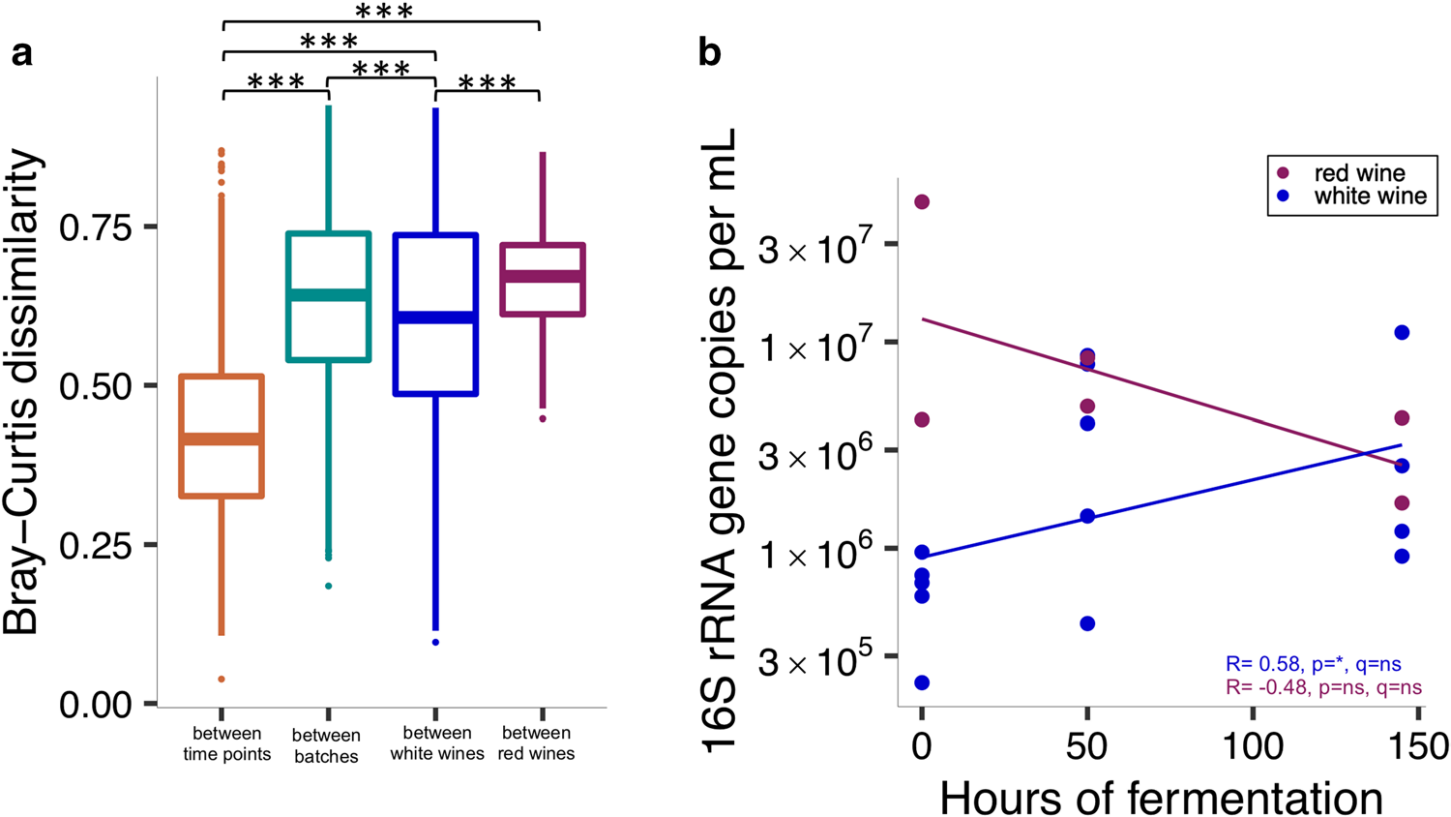
Bacterial microbiota changes during fermentation. Taxonomic microbiota compositions were compared between samples, based on Bray–Curtis dissimilarity, showing that samples from the same wine were more similar to each other and that red wine samples were more heterogeneous than white wine samples (**a**). Bacterial abundances of red and white wines showed opposing trends during fermentation, based on 16S rRNA gene copy numbers (**b**). Significance was calculated based on Wilcoxon rank-sum test (**a**) and Spearman’s rank correlation test (**b**), all corrected with the Benjamini–Hochberg procedure, with ns = not significant, *p/q < 0.05, ***p/q < 0.001.

While fermentation was generally associated with alterations in relative and absolute bacterial microbiota compositions in both red and white wines, changes varied between individual wine batches (Fig. 3a,b). Differences in taxonomic microbiota compositions were smaller between longitudinally collected samples from the same wine batch than between samples from distinct wine types or batches (Fig. 4a; Wilcoxon rank-sum test on Bray–Curtis dissimilarity). There was a trend towards a positive correlation of bacterial loads in white wines with the duration of the fermentation (Fig. 4b; p < 0.05, q = ns) and while both the relative and absolute abundance of *Tatumella* increased in all white wines during fermentation, differences were not significant (Supplementary Fig. S1a). Increased absolute abundances of the family *Bacillaceae* and the genus *Oenococcus* by the end of the fermentation period were the only significant changes observed across all red and white wines (Supplementary Fig. S1a), suggesting that pre-fermentation factors have a stronger impact on the bacterial wine microbiota than fermentation itself.

### Association of differences in microbial diversity with plant-derived read fractions and environmental stress factors

**Figure 5:**
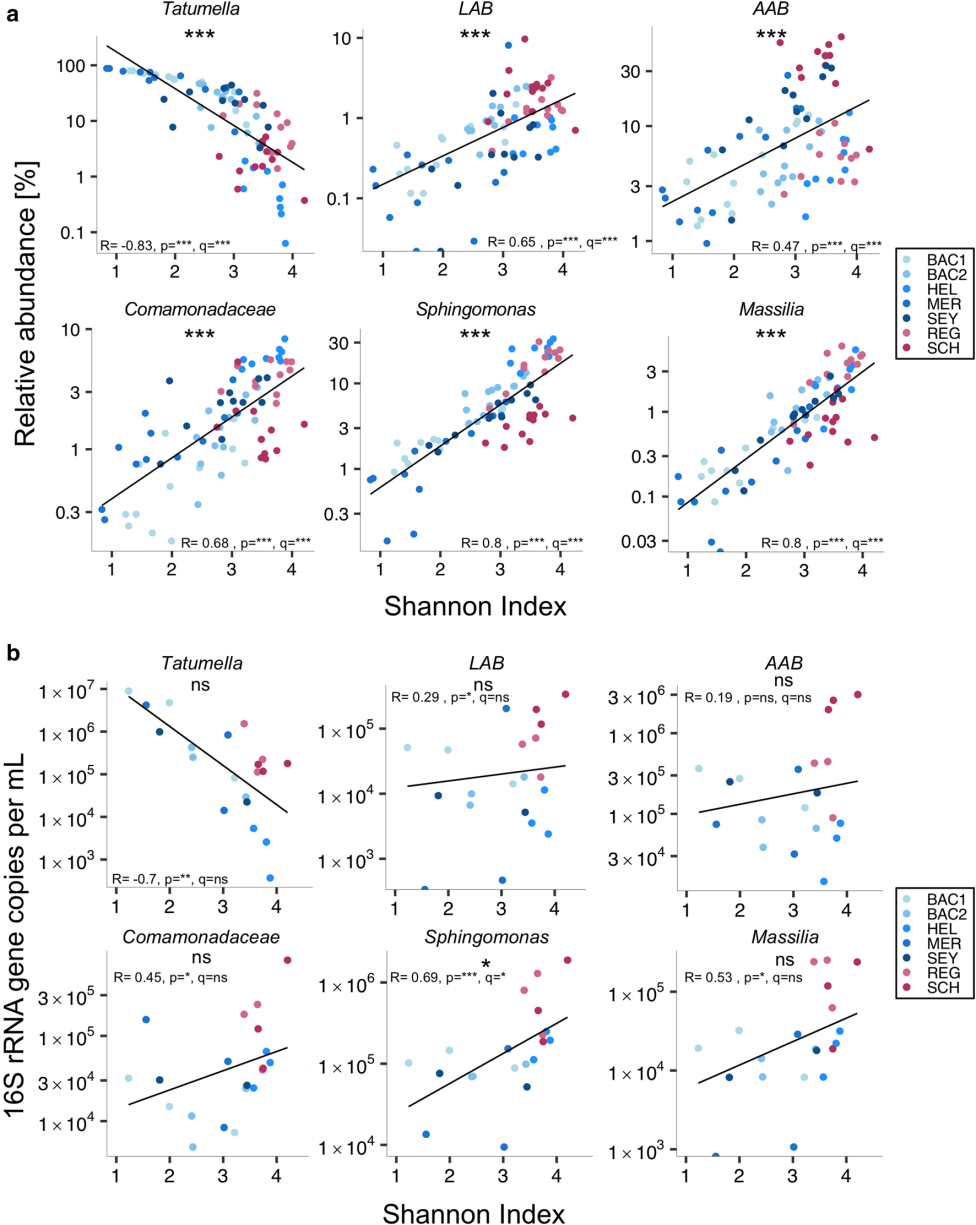
Relative and absolute abundances of *Tatumella*, acetic acid (AAB) and lactic acid bacteria (LAB) and other taxa were correlated with microbial diversity. Significance was calculated based on Spearman’s rank correlation test (**a**, **b**) corrected with the Benjamini–Hochberg procedure, with ns = not significant, *p/q < 0.05, ***p/q < 0.001.

Bacterial microbiota diversity differed between red and white wines (Fig. 1b) and considerably varied among white wines (Shannon diversity: 0.8–3.9), indicating that diversity was influenced by additional factors, independently of the wine type. Indeed, across all wine samples, alpha diversity was negatively correlated with the genus *Tatumella* and positively correlated with AAB, LAB, and CSM, including relative and absolute abundances (Fig. 5a,b).

**Figure 6:**
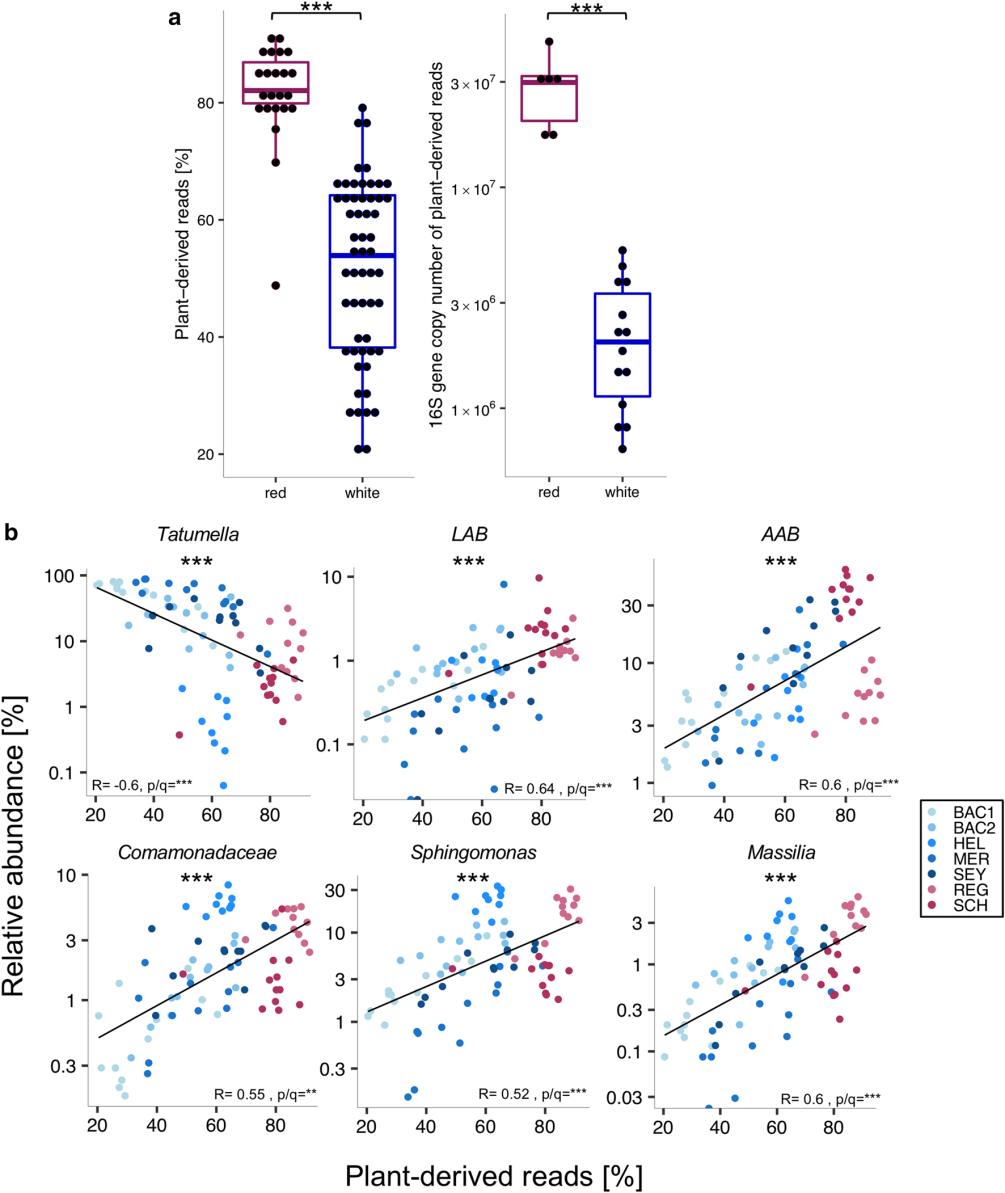
Plant-derived read fractions were correlated with the relative abundance of *Tatumella*, AAB, LAB and other taxa in wine. Significance was calculated based on Wilcoxon rank-sum test (**a**) and Spearman’s rank correlation test (**b**), all corrected with the Benjamini–Hochberg procedure, with ns = not significant, ***p/q < 0.001.

We hypothesized that differences between red and white wines that were involved in the processing of grapes, i.e. an additional mechanical destemming step for red but not white wine grapes, and in the fermentation process, i.e. a prolonged exposure of red wines to grape skins during fermentation, could increase microbial contributions from skin-attached environmental bacteria and result in higher bacterial microbiota diversity. We therefore compared plant-derived 16S rRNA amplicon read fractions from mitochondria and chloroplasts across all wine batches and time points (Supplementary Fig. S1b), as a potential marker for contact with grape skins and other plant tissues. As expected, we found higher relative and absolute contributions of plant-derived DNA in red compared to white wines (Fig. 6a). While there was no correlation between plant-derived read fractions and fermentation time (p = ns), the relative abundance of plant-derived reads varied substantially among white wines (Fig. 6a) and showed a positive association with bacterial microbiota diversity across red and white wine samples combined (Supplementary Fig. S2a). In addition, plant-derived read fractions were negatively correlated with the relative but not absolute abundance of *Tatumella* and positively correlated with the relative but not absolute abundances of AAB, LAB, and CSM (Fig. 6b and Supplementary Fig. S2c). The fact that both microbial diversity and plant-derived read fractions showed comparable associations with the same bacterial taxa could suggest that the mechanism that led to increased plant-derived reads was also responsible for increased microbial diversity. However, as correlations were stronger for microbial diversity, additional factors besides those that increased plant-derived reads would need to have contributed to microbial diversity in our wine.

To account for putative environmental influences on the bacterial wine microbiota, we compared wines based on infectious burdens of *B. cinerea* (Table 1). The rate of *B. cinerea* infection was positively correlated with microbial diversity in white wines but there was no significant correlation in red wines (Supplemental Fig. S2b). Again, as with plant-derived read fractions, the relative abundance of *Tatumella* was negatively and with CSM positively correlated with botrytis infection in white wines (Supplemental Fig. S3). Despite similar associations, botrytis infection rates and plant-derived read fractions were not significantly correlated with each other (data not shown), suggesting that they reflect independent mechanisms for increased bacterial microbiota diversity, reduced relative abundance of *Tatumella* and increased relative abundance of CSM in wine.

Of note, BAC2 grapevines, which had been experimentally treated with a combination of insect attractant and insecticide, showed a higher infestation with the insect fruit pest *Drosophila suzukii* than untreated BAC1 grapevines. We therefore compared wines from treated BAC2 and untreated BAC1 grapevines and found BAC2 samples to be characterized by increased plant-derived read fractions, as well as similar bacterial microbiota alterations as had been associated with botrytis infection, i.e. reduced relative abundance of *Tatumella* and increased relative abundances of *Sphingomonas* and *Massilia* (Supplemental Fig. S4).

## Discussion

Our study supports previous 16S rRNA gene amplicon sequencing-based reports of a complex and shifting bacterial microbiota in wine^11–15,17^, which we have expanded with complementary quantitative 16S rRNA gene abundance analysis. We show that red wine harbours a more diverse bacterial microbiota with a higher density of bacterial 16S rRNA genes per millilitre than white wine. The chromosomal copy number of the 16S rRNA gene is known to vary substantially between taxa^28^, but assuming on average four 16S rRNA genes per bacterial genome, we would expect red wines from our sample set to contain ~ 10^7^ bacterial genomes per mL or about tenfold more genomes than white wines. While not significant after correcting for multiple tests (q > 0.05), there was a clear trend towards increasing 16S rRNA gene copy numbers during white wine fermentation (p < 0.05), which resulted in comparable densities in red and white wines by the end of the one-week observation period. However, in our longitudinal analysis, fermentation time was associated with only moderate bacterial microbiota changes, as few bacterial taxa increased or decreased during fermentation and compositional differences between time points were small compared to those seen between wine types and batches. Calculating the fraction of taxon-specific 16S rRNA genes based on relative abundance values from the amplicon sequence analysis, we show that increases in overall 16S rRNA gene copy number density are mostly due to increased abundance of *Tatumella*, suggestive of active proliferation of this bacterial genus. Species from the genus *Tatumella* have been associated with fruits, including pineapple and mandarin orange, but also soil samples and even human patient specimens^28,29^. Whereas relative microbiota profiling alone would suggest reduced contributions of *Tatumella* to the red wine microbiota, the combination with quantitative microbiota profiling demonstrated comparable 16S rRNA gene densities for this genus, suggesting instead higher overall bacterial densities and additional contributions of other bacteria to the red wine microbiota. We identified three bacterial groups with higher absolute abundance in red compared to white wines, which are likely main contributors to the increased bacterial loads of red wines: acetic acid bacteria (AAB, from the genera *Acetobacter*, *Gluconacetobacter*, and *Gluconobacter*), lactic acid bacteria (from the genera *Lactobacillus*, *Oenococcus*, and *Lactococcus*) and a third group bacteria (CSM, including the family *Comamonadaceae* and the genera *Sphingomonas* and *Massilia*). The Gram-positive AAB are frequently found in wine but undesirable for wine production due their ability to efficiently convert ethanol to acetate, which is associated with spoilage, alters the wine aroma, and reduces its commercial value^30^. Grape skins have been suggested as a major source for AAB in wine, consistent with findings of reduced AAB abundance on berries washed by rain^31^. The LAB are physiologically more diverse and include *Oenococcus oeni*, which is typically responsible for the favourable malolactic fermentation^32^, but also *Lactobacillus* and *Pediococcus* species that can cause additional spontaneous fermentations with undesirable aromatic consequences^10,33^. The generally lower concentrations of LAB in wine, consistent in our samples, have been attributed to the mostly anaerobic lifestyles, suggesting competitive advantages for yeasts and AABs under the aerobic conditions of the grape^10^. *Sphingomonas* and *Massilia* species have been identified in rhizosphere and soil microbiomes^34,35^ and correspondingly, are frequently isolated from soil and water samples^36,37^. Thus, at least for AAB and bacteria from the CSM group an exogenous origin in wine is likely, e.g. through direct intake of grape skin-attached bacteria or through indirect environmental sampling of soil or water-associated bacteria that come into contact with grape skins. Martins et al. described the presence of a culturable, epiphytic bacterial microbiota on external grape berry and leaf surfaces, which overlapped with the CSM group from our study^38^, which could explain the increased relative and absolute contributions of these bacteria to the red wine microbiota.

As bacterial diversity and abundance of AAB, LAB and CSM in wine differed not only between red and white wines, but also among different grapevine cultivars and wine batches, we searched for additional factors and mechanisms that could explain variable exogenous contributions to the bacterial microbiota in wine. While we initially removed plant-derived 16S rRNA gene amplicon read fractions from chloroplasts and mitochondria as contaminants from our analysis, we later hypothesized that plant-derived read fractions could also represent biomarkers for wine exposure to plant-associated bacteria, particularly from grape skins. In line with this assumption, we found increased plant-derived read fractions in red wines and robust positive correlations with microbial diversity and the abundance of bacteria from putative exogenous sources, i.e. AAB, LAB and CSM, across all red and white wine samples. As the microbial ecology of grapes, including microbial burdens and species diversity is largely affected by the grape health status^10^, we also searched for factors associated with differences in plant-derived DNA contributions among white wines. We assumed that, by increasing interactions of grape juice with exogenous, skin-attached bacteria, interference with berry integrity before harvest could induce similar effects in white wines as extended grape skin contact during fermentation in red wines. Our collection of wines was exposed to two environmental stress factors with potential disruptive effects on grape skin integrity before harvest: (I) grapevines were differentially affected by infections with the fungal pathogen *Botrytis cinerea* and (II) as a consequence of the experimental application of an insect attractant/insecticide combination, Bacchus grapevines on adjacent sections of the vineyard were differentially exposed to infestations with the spotted fruit fly, *Drosophila suzukii*. *B. cinerea*, the causative agent of botrytis bunch rot in viticulture colonizes different plant organs and can penetrate the protective cuticle covering the grape epidermis^39–41^. Fermentations of botrytis-infected grapes have been shown to be enriched for bacteria and fungi and to contain increased microbial diversity and acetic acid bacteria concentrations^23^, in line with the higher bacterial microbiota diversity and abundance of AABs in those white wines from our collection that had a higher rate of botrytis infection. Similarly, Bokulich et al. showed that botrytized wine, i.e. fermentations from *B. cinerea*-infected but then dried, partially raisined grapes affected by “noble rot”, were characterized by high bacterial diversity^13^. *D. suzukii* could induce comparable bacterial microbiota effects, as these insects puncture the grape skin for oviposition^42^ and *D. suzukii* exposure has previously been associated with increased bacterial loads, most importantly of AABs^43^, in accordance with our findings from the direct comparison of the two Bacchus batches.

Our detailed qualitative and quantitative wine microbiota analysis supports the presence of a diverse bacterial microbiota in wine, which appears to be shaped by both endogenous and exogenous factors. On the one hand, comparable absolute abundance of the genus *Tatumella* in red and white wines, with a trend towards increasing density in white wines during fermentation, suggests a putative endogenous, grape juice-derived source of these bacteria in all wines, largely unaffected by external factors such as wine processing procedures and pathogen burdens before grape harvest. Robust positive associations of AAB, LAB and CSM with markers of plant tissue contributions and pre-harvest pathogen burdens, on the other hand, suggest a putative exogenous, grape skin-derived source of these bacteria in all wines, resulting in increased loads in red wines and wines affected by *B. cinerae* or *D. suzukii*. Additional studies will be needed to further our understanding of endogenous and exogenous contributions to the wine microbiota, including larger sample sets that span a wider variety of wine types, cultivars, and environmental conditions.

The specific metabolic contributions of those bacteria classified here as of putative exogenous origin to the extended secondary plant metabolite spectrum in red wines remain largely unknown. However, it is conceivable that exogenous factors, ascribed to plant tissue contributions, botrytis and fruit fly infestation in our analysis, could be leveraged to deliberately increase, modify, or expand the aromatic quality of wine. In fact, botrytized white wines would represent an example, as well as the production of “orange”, or skin-contact, white wines that are characterized by increased phenolic concentrations of antioxidant potential^44–47^.

## Methods

### Grapes, metadata and sample collection

All grapevines, including the two red wine cultivars Regent (REG) and Schwarzriesling (SCH) and the four white wine cultivars Merzling (MER), Seyval (SEY), Helios (HEL), and Bacchus (BAC) were grown on the vineyards of the University of Hohenheim, Stuttgart, Germany in 2015. Bacchus grapevines were divided into two separate groups: plants from the first group did not receive special treatment (BAC1), whereas plants from the second (BAC2) underwent treatment with a combination of insect attractant and insecticide (Combi-protec with insecticide SpinTor, Belchim Crop Protection, Belgium), which was associated with higher infestation of spotted wing drosophila, *Drosophila suzukii*, in the BAC2 grapevine. All grapes were harvested within three weeks (September/October 2015). Within two hours after collection grapes were processed for red and white wine production, with grapes intended for red wine production undergoing a mechanical destemming before crushing. The first samples were collected before and after the grape must was inoculated with a commercial *Saccharomyces cerevisiae* yeast starter culture (NT2000/NT50/NT112, Oenobrands SAS, France). Additional samples were collected three times per day for the first three days and daily for the remaining days during the first week of fermentation. All samples were immediately stored at − 80 °C until further processing.

### Oenological parameters

Oenological parameters were measured as previously described^48^. Total soluble solids (°Brix) of wine were determined using a refractometer (Opton, Zeiss, Germany) and total acid and pH by titration (TiroLine easy, Schott, Germany). High performance liquid chromatography (Merck-Hitachi, Germany) was carried out to determine wine alcohol contents (flow rate: 0.5 mL/min; detection at 210 nm), using a Rezex ROA-Organic Acid H + (8%), LC Column 300 × 7.8 mm, Ea (Phenomenex, Germany) in combination with a SecurityGuard Cartridge, Carbo-H 4 × 3.0 mm pre-column (Phenomenex, Germany).

### DNA extraction

Metagenomic DNA of all samples was isolated using a previously described method^49^ from our group, which combines both enzymatic digestion and mechanical disruption by bead beating. In brief, aliquots of 500 µL per sample were thawed on ice and centrifuged for 15 min at 4,000×*g*. The pellet was washed in 1 mL PBS, centrifuged for 5 min at 8,000×*g*, resuspended in 700 µL PBS and transferred to a Lysis B Matrix tube (MP Biochemicals, France) for bead beating. Enzymatic cell lysis (lysozyme, mutanolysin, lysostaphin, proteinase K and RNase) was initiated as described in the method above. The resulting cell lysate was processed with the ZR Fecal DNA mini-prep kit (Zymo Research, USA) according to the manufacturer’s recommendation and eluted in ultra-pure water. As controls, blank DNA extractions and extractions from the yeast starter cultures were included and further processed along with the wine samples. The DNA was stored at -20 °C until further processing.

### 16S rRNA gene amplification and sequencing

Hypervariable region V4 of the 16S rRNA gene was amplified from metagenomic DNA via PCR using Phusion High-Fidelity PCR Master Mix (Thermo Fisher Scientific, USA) and Golay-barcoded primers 515F and 806R adapted from Caporaso et al.^50^ and additionally modified by adding internal spacers of 0 to 7 bp, adapted from Fadrosh et al.^51^. Primer, barcode and spacer sequences are listed in Supplementary Table S5. The PCR reaction contained 10 µL of 2 × Phusion Master Mix (Thermo Fisher Scientific, USA), 0.8 µL of each primer (final concentration 0.4 µM), 0.6 µL dimethyl sulfoxide (DMSO), and 7.8µL template DNA. PCR amplifications were carried out as described previously^52^, with an initialization step at 98 °C for 2 min, followed by 30 cycles of 98 °C for 10 s, 52 °C for 15 s and 72 °C for 15 s, and a final extension at 72 °C for 5 min. The SequalPrep normalization plate kit 96 (Thermo Fisher Scientific, USA) was used to select equimolar PCR product amounts, which were subsequently pooled and concentrated with the DNA Clean and Concentrator 5 kit (Zymo Research, USA). Sequencing libraries were generated with the TruSeq Nano DNA LT Library Prep kit (Illumina, USA) for sequencing on the Illumina MiSeq platform (MiSeq Reagent Kit v3, 600 cycles, Illumina, USA) at the University of Hohenheim, following the manufacturer’s recommendations.

### Quantitative 16S rRNA gene amplification

Metagenomic DNA was diluted to a concentration of approximately 1 ng/µL, of which 2 µL were used as template for quantitative PCR amplification of the universal bacterial 16S rRNA gene with the Femto bacterial DNA quantification kit (Zymo Research, USA) according to the manufacturer’s recommendations. All reactions were carried out in duplicates. Genomic DNA from *E. coli* strain JM109 (Zymo Research, USA) was used as an internal standard to estimate bacterial 16S rRNA gene copy numbers. The PCR was run on a CFX96 Touch real-time detection system (Bio-Rad, USA). Samples were considered non-amplified if the quantification cycle (Cq) value was greater than 39. Samples with quantification cycle (Cq) values greater than 39 were discarded; average Cq values of duplicates were calculated for each sample and used to determine 16S rRNA gene copy numbers. Bacterial 16S rRNA gene copy numbers were calculated for each sample as concentrations per 1 mL of wine. Taxon-specific absolute abundances were determined by multiplying relative abundance values from the 16S rRNA gene amplicon sequence analysis with total 16S rRNA gene copy numbers (see Supplementary Table S3).

### Microbiota analysis and statistical methods

Pre-processing of raw sequence data was performed with QIIME v1.9.1^53,54^, cutadapt v1.10^55^ and custom Python scripts, including trimming of spacer and primer sequences, merging of raw paired end reads using bbmerge v9.02^56^, barcode extraction and demultiplexing of samples. The processed sequences were imported into QIIME2 v2018.2. OTUs were generated using open-reference OTU picking with a similarity threshold of 97% and classified with the q2-feature-classifier^57^ against the Greengenes database v13_8 (greengenes.lbl.gov). Chimera checking was performed with vsearch^58^, as part of the QIIME2 pipeline. OTU’s taxonomically assigned to chloroplast and mitochondria were searched against the NCBI nucleotide database by BLAST^59^, which identified grapevine, *Vitis vinifera*, and its grafted rootstock, *Vitis riparia*, as closest matching sequences. These sequence reads accounted for 58% of the sequences and were removed from the analysis, unless noted otherwise. For diversity analyses, all samples were rarefied to 3,500 reads per sample. Detailed information about sequenced OTU’s and taxonomic assignment per sample is listed in Supplementary Tables S6–S13. Unless indicated differently, the final dataset consisted of n = 56 white and n = 23 red wine samples for sequenced data. For qPCR data, the final set consisted of n = 14 white and n = 6 red wine samples. Statistical testing and data visualization were carried out in R (www.R-project.org/), using the packages *vegan*, *biomformat*, *nortest* and *ALDEx2*^60^. All parameters were tested for normal distribution with Anderson–Darling and Shapiro–Wilk tests. Features were filtered to only include OTUs with a relative abundance of ≥ 1% in all samples or of ≥ 5% in at least one sample. Not normally distributed data were analysed using non-parametric tests. For group comparisons, pairwise Wilcoxon rank sum test was used, for correlation analyses Spearman's rank correlation tests and for differential abundance analyses ANOVA-Like Differential Expression (ALDEx) Analysis^60^ with 128 Dirichlet Monte-Carlo Instances (DMC). All tests were corrected with the Benjamini–Hochberg procedure, based on the number of features (n = 79). Comparisons of dissimilarities between communities were done by analysis of similarities (ANOSIM)^61^, with the number of permutations set to 999, of the Bray–Curtis dissimilarity. Significance levels were determined as p > 0.05 (ns), p < 0.05 (*), p < 0.01 (**) and p < 0.001 (***). Unless indicated, mean values are presented with standard error of means (SEM). Detailed information about all bioinformatic scripts and commands used for the analysis is provided in Supplementary Table S13.

## Supplementary information

Supplementary figures

Supplementary tables

## Data Availability

Pre-processed, trimmed and merged paired-end read contigs have been deposited in the European Nucleotide Archive under primary accession number PRJEB37054 (secondary accession number ERP120343).
