# Supplementary material for: Bacterial microbiota diversity and composition in red and white wines correlate with plant-derived DNA contributions and botrytis infection
Source: Sci Rep. 2020 Aug 14;10:13828. doi: 10.1038/s41598-020-70535-8 (PMC7427798; doi:10.1038/s41598-020-70535-8)
Supplement: Supplementary file 1 — Supplementary figures [file 41598_2020_70535_MOESM1_ESM.pdf]

Bacterial microbiota diversity and composition in red and white wines correlate with plant-derived DNA contributions and botrytis infection

Alena M. Bubeck[1], Lena Preiss[1], Anna Jung[1], Elisabeth Dörner[1], Daniel Podlesny[1], Marija Kulis[1], Cynthia Maddox[1,3,4], Cesar Arze[1,3,5], Christian Zörb[2], Nikolaus Merkt[2], W. Florian Fricke[1,3]*

1Dept. of Microbiome Research and Applied Bioinformatics, Institute for Nutritional Sciences, University of Hohenheim, Stuttgart, Germany

2Dept. of Plant Quality and Viticulture, Institute of Crop Science, University of Hohenheim, Stuttgart, Germany

3Institute for Genome Sciences, University of Maryland School of Medicine, Baltimore, MD, USA

Current affiliations

4Personal Genome Diagnostics, Baltimore, MD, USA

5Ring Therapeutics, Cambridge, MA, USA

⋆Corresponding author: w.florian.fricke@uni-hohenheim.de

Supplementary Figures

Fig. S1a

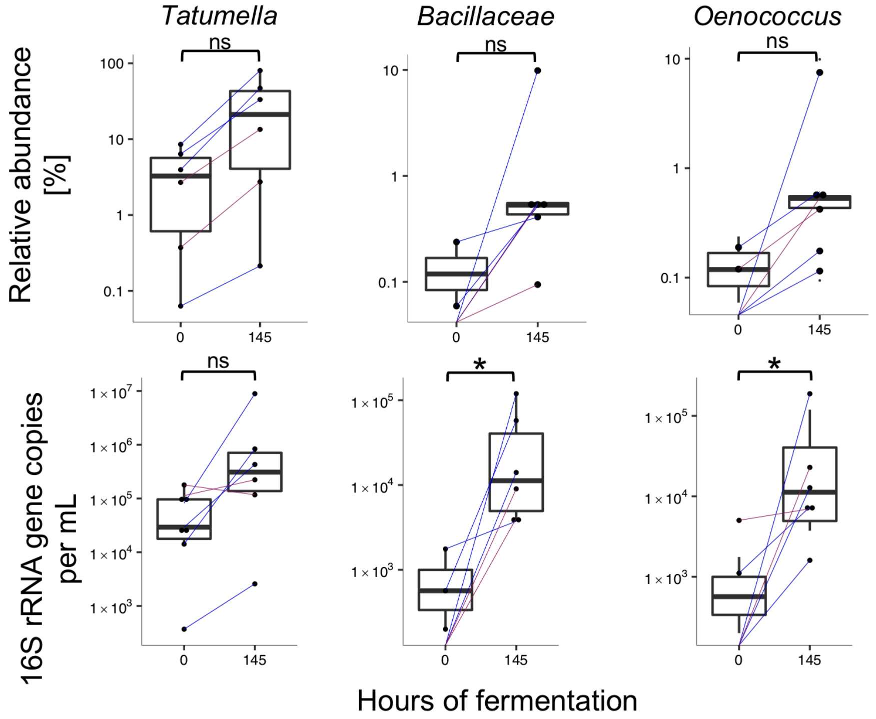

Fig. S1b

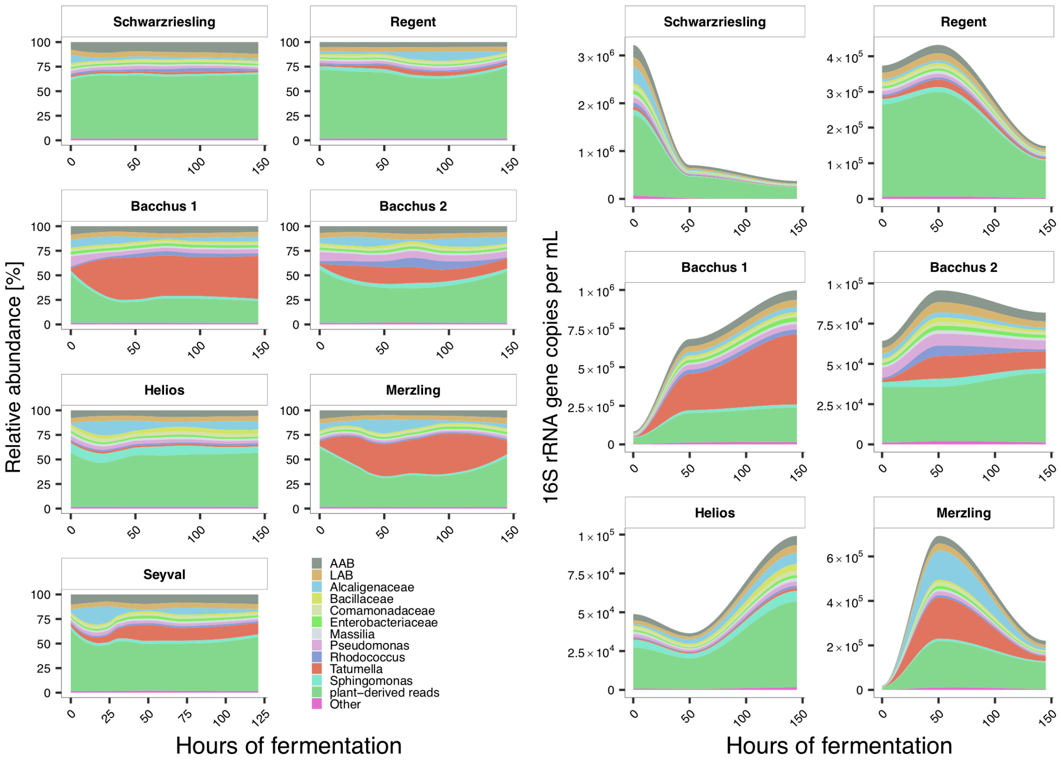

Figure S1: Relative (a) and absolute (b) bacterial microbiota composition changes during fermentation, including plant-derived reads. Neither relative nor absolute abundance of *Tatumella* was altered during fermentation, as opposed to the family *Bacillaceae* and the genus *Oenococcus* (a). Figure (b) shows the eleven most abundant

assigned taxa on genus level and all OTU's assigned to plant-derived reads as "other". (a) Significance was calculated based on ALDEx analysis with 128 DMCs and Wilcoxon rank-sum test both corrected with the Benjamini-Hochberg procedure, with ns=not significant, *p/q<0.05 and ***p/q<0.001. Effect sizes for ALDEx analysis were *Tatumella*= 0.61, *Bacillaceae*= 1.2 and *Oenococcus*= 1.31. (b) Locally weighted regression was used to smooth relative abundances over time.

Fig. S2a

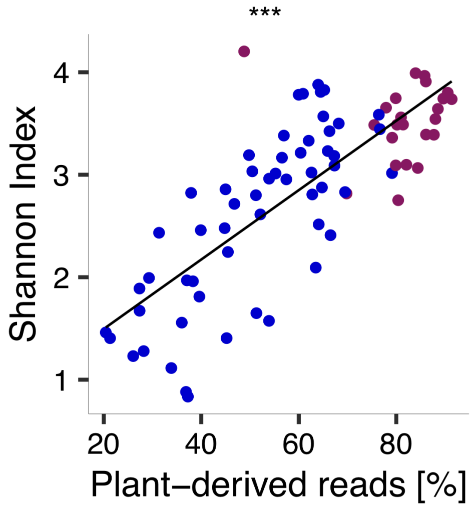

Fig. S2b

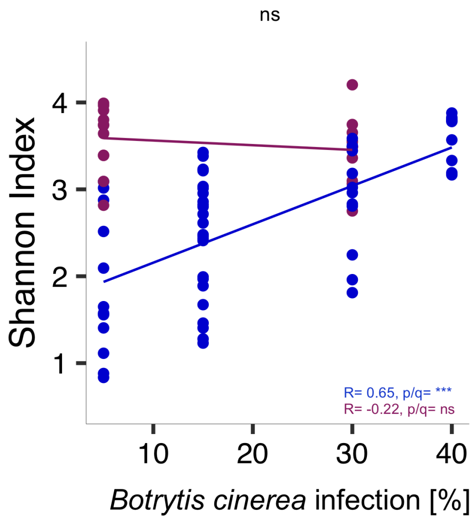

Fig. S2c

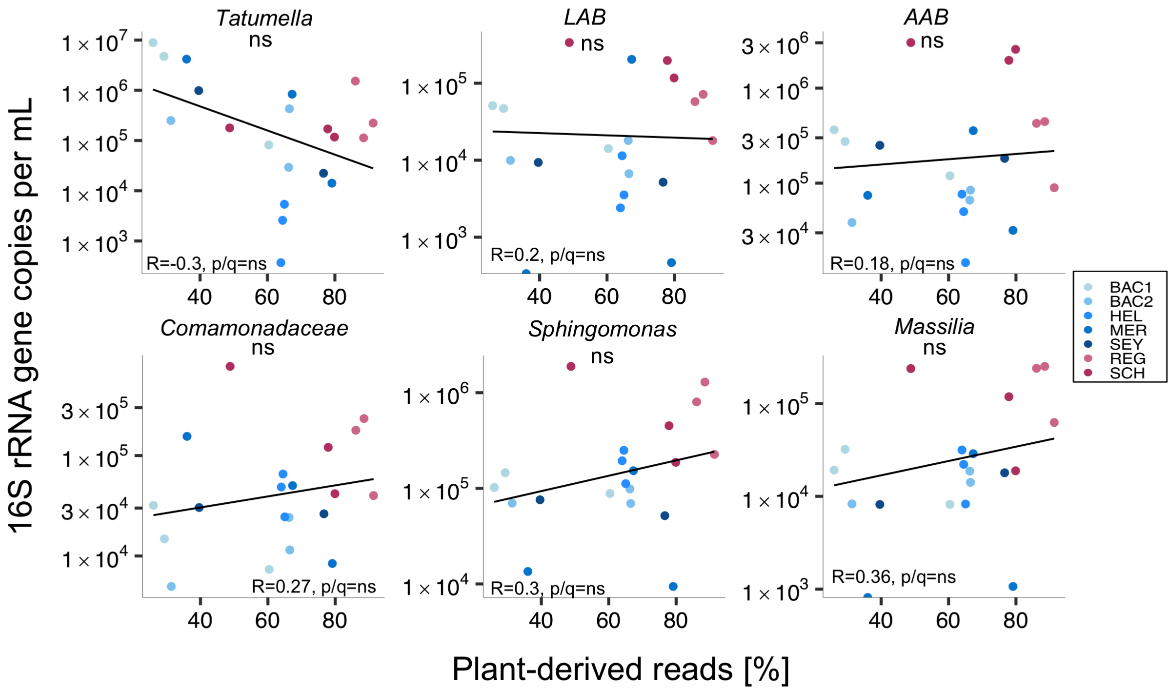

Figure S2: Microbial diversity (a,b) correlated with external factors such as plant-derived read fractions and *Botrytis cinerea* infections. Absolute abundances of *Tatumella*, LAB, AAB and CSM did not correlate with plant-derived read fractions in red and white wines (c). Significance was calculated based on Spearman's rank correlation test corrected with the Benjamini-Hochberg procedure, with ns=not significant, ***p/q<0.001.

Fig. S3

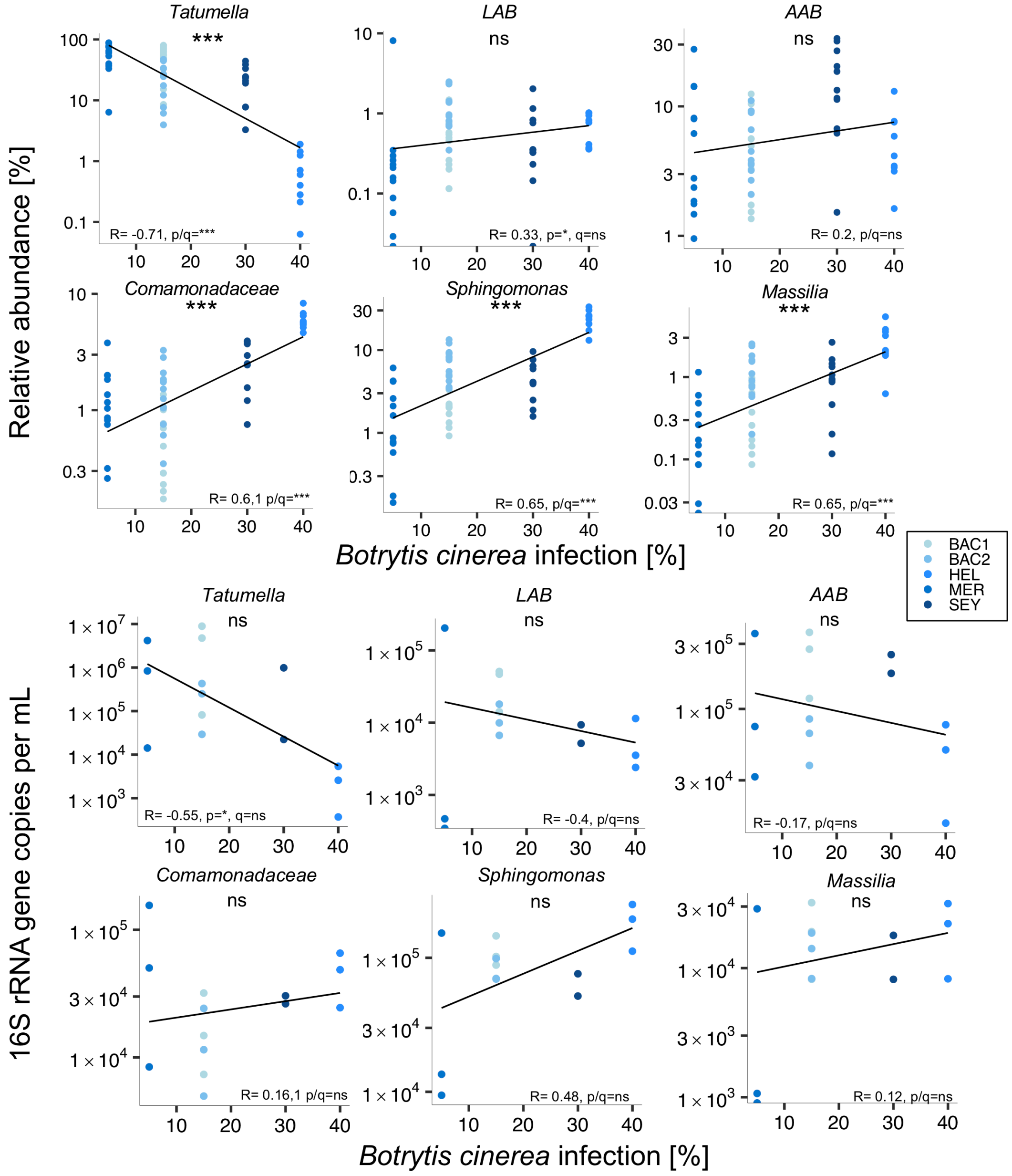

Figure S3: Botrytis infection was correlated with the relative abundance of *Tatumella*, and other taxa in wine. Significance was calculated based on Spearman's rank correlation test corrected with the Benjamini-Hochberg procedure, with ns=not significant and ***p/q<0.001.

Fig. S4

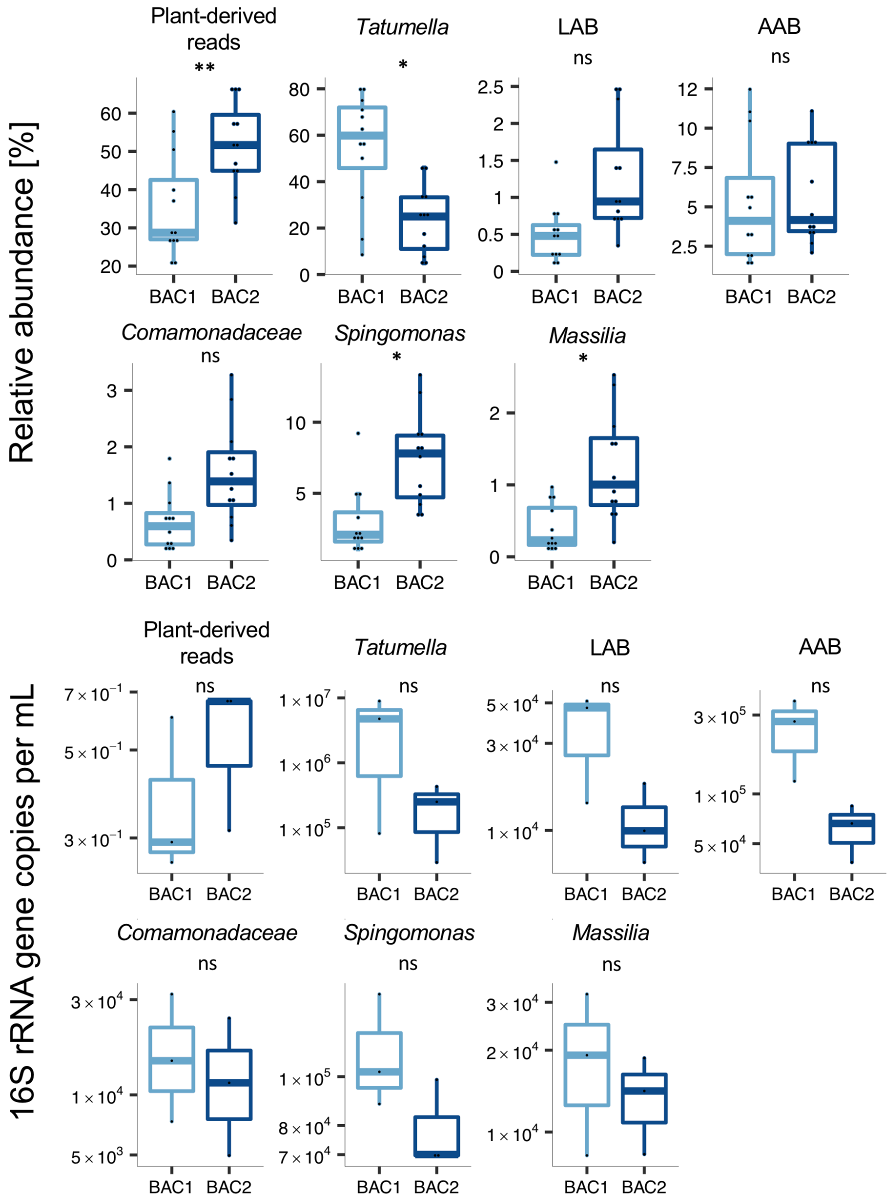

Figure S4: The relative abundance of *Tatumella* was reduced and of *Sphingomonas* and *Massilia* increased in BAC2 compared to BAC1. Significance was calculated based on ALDEx analysis with 128 DMCs and Wilcoxon rank-sum test both corrected with the Benjamini-Hochberg procedure, with ns=not significant, *p/q<0.05, **p/q<0.01. Effect sizes for ALDEx analyses were *Tatumella*= -0.93, LAB= 0.65, AAB= -0.45, *Comamonadaceae*= 0.41, *Sphingomonas*=0.9, *Massilia*=0.8 (a).
